# Georgia and the Black Sea: Risks, Resilience and Opportunities

**DOI:** 10.1007/s12399-022-00913-9

**Published:** 2022-09-12

**Authors:** Elguja Khokrishvili, Bidzina Lebanidze

**Affiliations:** 1 Free University of Berlin, Berlin, Germany; 2 School of International Economics, Tbilisi State University, Tbilisi, Georgia; 3 University of Jena, Jena, Germany; 4 Ilia State University, Tbilisi, Georgia

**Keywords:** Georgia, Black Sea connectivity, EU, Security, Economy, Resilience, Georgien, Schwarzes Meer, EU, Sicherheit, Wirtschaft, Resilienz

## Abstract

By unpacking major views of and dynamics towards the Black Sea region from the Georgian perspective, this analysis addresses two questions: What are Georgia’s key perceptions of the Black Sea region? And which role does the Black Sea play in strengthening Georgia’s economic and security resilience in a quickly changing environment marked by geopolitical and geo-economic competition? The article concludes with thoughts on the region’s future and the role of the EU, Georgia’s closest partner, in it.

## Abstract (de)

Diese Analyse untersucht, welche Rolle die Schwarzmeerregion in der georgischen Sicherheits- und Wirtschaftspolitik spielt. Der Fokus liegt auf zwei Fragen: Was sind die wichtigsten Wahrnehmungen Georgiens der Schwarzmeerregion? Welche Rolle spielt das Schwarze Meer bei der Stärkung der wirtschaftlichen und sicherheitsbezogenen Widerstandsfähigkeit Georgiens in einem sich schnell verändernden Umfeld, das von geopolitischem und geoökonomischem Wettbewerb geprägt ist? Der Artikel schließt mit Überlegungen zur Zukunft der Region und der Rolle der EU als engstem Partner von Georgien.

## Source of risks and opportunities: Georgia’s perceptions of the Black Sea Area

In the perception of Georgia’s political elites, the Black Sea Area is associated with both risks and opportunities. Georgia associates the Black Sea with several images.

First, for Georgia the Black Sea region (BSR) has a function of a transit point with yet unfulfilled potential. As a link between Europe and Asia, the BSR has always been of great geopolitical and geoeconomic importance as a transit point on global East-West and North-South trade routes. Strategic documents of Georgia underline the importance of the Black Sea as a source of economic exchange, investments, and tourism. Moreover, Georgia considers its littoral state status as conducive of regional cooperation and peacemaking in the volatile South Caucasus region. According to Georgia’s National Security Strategy, “[t]he potential of Georgia as a Black Sea littoral state is a supporting factor for the development of multilateral cooperation [among the three South Caucasus states]” (MOD Georgia 2011, p. 17). However, for the past decade and a half, the Black Sea region has not developed into a vibrant zone for trade, transport, energy, or cultural exchange. Instead, much of the region’s potential was lost as it has become a scene of struggle for dominance and competing geopolitical interests. This is the second image which informs and shapes Georgia’s regional perceptions.

Ongoing geopolitical competition, dominated by Russian geopolitical presence, is another prevailing issue in the Black Sea region. Georgia considers the Black Sea as a potential source of danger to its national security and stability. With regard to Russia’s challenge in the Black Sea, military conflicts in Georgia and Ukraine are considered as constituting parts of the same process which poses serious threats to the peace and economic development in the Black Sea (MFA Georgia 2019). Russia’s aggression against Georgia and Ukraine allows it to exponentially increase its military presence in the BSR, and to establish an effective platform for power projection.

Next to geopolitical aspects and risks emanating from Russia’s heavy presence, the Black Sea is also considered an arena of geoeconomic competition. New opportunities start to emerge as political, security, trade, and economic interests of several actors in the Black Sea region are increasing. Besides the littoral states these are the EU and NATO, the USA and China. For Georgia, this means more involvement in potential economic, energy, and connectivity projects. From this perspective, Georgia could benefit from an increased presence of its Western partners which would have “a positive impact on regional security and economic development allowing the wider Black Sea region to play its historical role of becoming a valuable geo-economic hub” (MOD Georgia 2021, p. 14).

The Black Sea is also considered as a geographic compass and the important bridge towards the EU and the NATO. Full membership in both Western organizations remains the key objective of Georgia’s foreign policy and the Black Sea is the only area offering Georgia direct geographic links to their member states: Turkey, Bulgaria and Romania. Moreover, in the context of Georgia’s European integration, belonging to the Black Sea area seems to also be connected to the rebuilding of Georgia’s national identity. This identity reformulation effort seeks to underplay sub-regional aspects and highlighting Georgia’s trans-regional linkages. For example, political elites in Georgia have long been trying to disassociate the country from the South Caucasus region and associate it more with the Eastern European community, politically and culturally. The image of being a Black Sea country seems to be helpful in this regard as it provides symbolic and physical connection with other littoral Eastern European states. For instance, the 2019–2022 Foreign Policy Strategy of Georgia mentions the “Eastern-European-Black Sea region” which also includes Georgia (MFA Georgia 2019, p. 2). According to the Georgian National Security Concept, “as a Black Sea and Southeast European country, Georgia is part of Europe geographically, politically, and culturally; yet it was cut off from its natural course of development by historical cataclysms” (MOD Georgia 2011, p. 15). On this background, Georgia’s MOD Vision 2030 sees Russia’s coercive actions as continuous attempts to undermine “Georgia’s chosen course towards unification with the Western, European Family” (MOD Georgia 2021, p. 14). The civilizational dimension of Georgia’s European choice is further echoed by the latest Foreign Policy Strategy. According to the document, membership of EU and NATO is based on “civilizational choice of Georgia, is a matter of a broad societal consensus and guaranteed by constitution of Georgia” (MFA Georgia 2019, p. 4). On Georgia’s insistence, the EU-Georgia Association Agreement also recognized Georgia as “an Eastern European country”^1^ being committed to implementing and promoting the “common values on which the EU is built” (Official Journal of the European Union 2014, p. 5).

## The role of the Black Sea Area in Georgia’s security

The Black Sea Area has a significant impact on Georgia’s security, stability, and development. At the same time, its role can be quite ambivalent – it can act both as a spoiler and facilitator of Georgia’s security and stability. On the one hand, the Black Sea Area has recently turned into the main geographic space of Russia’s geopolitical assertiveness which both directly and indirectly undermines Georgia’s national sovereignty and threatens its statehood and foreign policy priorities. Direct negative impact is generated by Russian occupation of Abkhazia, and to lesser extent of South Ossetia, and resulting dominance of Russian navy in Georgia’s littoral waters. Georgia is especially vulnerable towards the military risks emanating from Russia’s heavy naval and military presence in the Black Sea Area. It is worth recalling that the Black Sea shore was one of the key scene’s during the 2008 war when Russian navy decimated Georgia’s fleet and kept a maritime cordon blocking access to the port of Poti and the entire Georgian coast (Institute for War and Peace Reporting 2013). Russia holds more than 8000 military personnel and modern military equipment, including S‑300 air defense system in Abkhazia and South Ossetia (Batashvili 2017). Russia also maintains naval military presence in the strategically important port of Ochamchire located in Abkhazia. Tbilisi itself does not have any naval military presence after the 2008 Russia-Georgia War. Its small coast guard has no chance against Russian navy. From sea, Georgia is virtually undefended in case of Russia’s recurring incursion (Kuimova and Wezeman 2018).

Indirect negative impacts also stem from Russia’s occupation of Crimea and the military conflict in Eastern Ukraine. Russia’s long-term strategy seems to be to transform “the Black Sea into an anti-access/area denial zone” (Seskuria 2020) which threatens Georgia’s transit hub potential and its connectivity strategy to reach out to the outside world including NATO and EU partners via the Black Sea. The 2020 war between Armenia and Azerbaijan and the deployment of Russian peacekeeping forces in Nagorno Karabagh region added another layer to Georgia’s deteriorated security. The conflict also highlighted Turkey’s geopolitical assertiveness which can act as a counterbalance to Russia’s regional dominance amid the fading Western influence (Georgian Institute for Politics 2021).

After the 2008 Russia-Georgia war it became clear that NATO and EU memberships were on hold and the Western actors were not ready to bring Georgia under their military and security umbrella. However, deepening ties with both organisations in order to hedge the security risks in Georgia and the wider Black Sea region continued below the formal membership threshold. After the war the EU sent the European Union Monitoring Mission (EUMM) to observe the peace between the conflict parties, which to this day is the only international mission in Georgia providing a minimal deterrent against Russia (Kakachia et al. 2020). Georgia’s practical approximation to NATO structures also accelerated after the 2008 war and resulted in Georgia becoming a NATO Enhanced Opportunities Partner country in 2016, a status which provides “all of the privileges that alliance members receive except for the collective security umbrella” (Paul and Andguladze 2018). Closer military ties to NATO were also accompanied by strengthening bilateral partnerships with Georgia’s key geopolitical partners – including the US and Black Sea neighbor Turkey. In 2021 the US and Georgia signed a new bilateral U.S.-Georgia security cooperation initiative, the Georgia Defense and Deterrence Enhancement Initiative, aimed at further modernization and development of Georgian military (US Embassy in Georgia 2021).

Finally, Georgia also stepped up its security partnership within the newly established Associated Trio group consisting of Ukraine, Moldova, and Georgia. The three countries seem to share the same threat perceptions towards Russia and foreign policy priorities. Therefore, the Black Sea Security and cooperation in the Black Sea Area have become one of the key priorities of the newly established group. In the communique of the 2021 EU Eastern Partnership (EaP) summit, the Council of the EU acknowledged the Trio as a distinct format and committed itself to exploring options for enhanced sectoral cooperation (Council of the European Union 2021).

Last but not least, Georgia also has been trying to balance Russia’s geopolitical assertiveness by normalising relations with Moscow. After the 2012 power change in Georgia, relations between both countries have intensified somewhat, especially in areas of trade, tourism, investments, and economic exchanges (Kakachia and Lebanidze 2019). However, Georgia’s approach not to irritate Russia soon hit its limits due to public discontent but also a lack of progress in conflict areas and security-related issues (Kakachia and Lebanidze 2019). To summarize, Georgia has tried but so far failed to establish a meaningful deterrent towards Russia-related security risks in the Black Sea area making the country vulnerable to a variety of hostile tactics incoming from the northern neighbor.

## The role of the Black Sea in Georgia’s economic strategy

Georgia’s relationship with Black Sea countries is not only about its security concerns. To a big extent they are driven by trade in goods and Georgia’s vision to become an East-West transport hub. To establish itself as a hub linking East and West, Georgia is primarily pursuing – with various degrees of success – two series of measures: creating a network of free-trade agreements and expanding the country’s road, rail, and sea traffic infrastructure. Georgia is an open economy and offers liberal regulations for foreign investors. Over the last two decades the country has signed several strategic trade agreements with Black Sea states and extra-regional players, including the Deep and Comprehensive Free Trade Agreement with the EU, free trade agreements (FTAs) with the Commonwealth of Independent States, Turkey, EFTA countries, China and Hong Kong. It also enjoys preferential trade regimes within its relations with the US, Canada, and Japan. According to one source, “Currently, products made in Georgia have access to one-third of the world’s consumer market through free trade regimes which reach more than 2.3 billion consumers in total” (Agenda.ge 2020). Particularly successful was the outreach to the Chinese market, Georgia’s exports grew from a meagre US$ 25.6 million in 2012 to US$ 615.5 million in 2021. Thus, China became Georgia’s second biggest trading partner by exports, after the EU-27 (US$ 717 million) (GeoStat 2022).

Despite successes in accessing distant markets, the Black See region still remains an important export market and significant source of imports for Georgia. In 2021, under the country’s top ten single export partners were four Black Sea countries, led by Russia (14.4% of exports), followed by Turkey (7.6%), Ukraine (7.2%), Bulgaria (6%) and Romania (0.7%). Also, Georgia’s two biggest single import partners were from the Black See region, namely Turkey at 18.1% of the total import and Russia at 10.2%, followed by Ukraine at 4.5% in the seventh place and Bulgaria and Romania with 1.7% each of them (Ibid).

The Black Sea plays an important role in Georgia’s outreach to global markets but also in facilitating international trade. Georgia’s Black Sea ports are important assets and serve mainly transit traffic in and out of the region. The share of maritime transport trade (US$ 4.5 billion) in Georgia’s foreign trade turnover (US$ 11.3 billion) is about 40%, while the rest comes by road (42%), rail (6.7%), air (7.6%) and other types of transport^2^.

In 2020, the total volume of goods handled by the ports amounted to 16.9 million tons (approximately 8 million tons was oil and oil products), which is slightly less than in previous year and probably can be explained by a Covid related slowdown in trade. Georgia’s own export and import needs through the ports did not exceed 4 to 5 million tons, so a large part of the goods handled by Georgian ports were transit goods (Tsereteli 2021). In 2019, the total transshipment in Georgian ports increased by 39.5% compared to the previous year (after 15% annual growth in 2018). This was mainly due to the growing demand for imports in the South Caucasus region and Central Asia, which is being met through Georgian ports. The maximum capacity for processing containers in Georgian ports is 750,000 TEU and in 2019, the total number of containers processed by Georgian seaports amounted to 647,816 TEU, which was 42.7% more than in 2018 (Business Media Georgia 2020).

Georgia currently relies on its two main ports (Poti and Batumi) and the two oil terminals (Kulevi and Supsa) (Brattberg et al. 2021). Poti is the largest port in Georgia, occupying about 80% of the volume processed in Georgian ports (12 million tons). Poti is mostly servicing bulk cargos and containers and it transhipped 7.4 million tons in 2020 (Tsereteli 2021). Batumi has a capacity of up to 18 million tons, but up to 15 million tons of capacity is for liquid cargos, mostly oil products. Supsa, is connected to the Baku-Supsa pipeline and Kulevi (Tsereteli 2021).

Georgian ports in Poti and Batumi “are not deep enough for Panamax container ships”, which means that cargo entering or leaving Georgia “has to be transferred to other ships in Istanbul or Constanta”, causing delays (Sanders 2021). For this reason, in 2017 the Georgian government chose through the tender Anaklia Development Consortium (ADC) to build a new deep-water port and a free industrial zone (FIZ) at Anaklia, a few kilometres away from the Russian occupied Abkhazia region. The project was aiming to open the port to shipping in 2020, with a planned annual handling capacity of 100 million tons. of cargo (Anaklia Development Consortium 2022), which could not only have strengthened the country’s position on the maritime Silk Road, but would have also significantly increased Georgia’s importance to BSR. The project was suspended in 2020 as the government was accused of having a vested interest in the other project in Poti (Civil Georgia 2021). In January 2022, the Pace Group, a Georgian-American transport company, unveiled a new Pace terminal in Poti port which will handle 2.5 million tons. of bulk cargo and 100,000 TEU of containers, but much less than those planned for Anaklia (Civil Georgia 2022). Although the Anaklia project also appears to be back on the political agenda, the project is still on hold.

The Black Sea region also is an important international route for energy flows with Georgia being a key transit country. There are currently several key transit energy infrastructure elements with international significance. These include two oil pipelines: Baku-Supsa pipeline connecting the Azerbaijan section of the Caspian Sea to the Georgian Black Sea port of Supsa, and Baku-Tbilisi-Ceyhan pipeline supplying Caspian crude oil to Turkey’s Mediterranean port of Ceyhan (Tsereteli 2020). Another infrastructure network of international importance is the South Caucasus Gas Pipeline (SCP) which exports natural gas from the Shah Deniz field in the Caspian Sea in Azerbaijan, to Georgia and Turkey (Tsereteli 2020). This pipeline system has provided Georgia with a much-needed alternative to Russian oil and natural gas supplies, cementing the country’s energy security.^3^

The South Caucasus Gas Pipeline (SCG) has become the basis for the larger pipeline system the Southern Gas Corridor (SGC). The Southern Gas Corridor is linking a SCG with the Trans-Anatolian Pipeline – TANAP (connects the Turkish border to Greece and Georgia) and the Trans-Adriatic Pipeline – TAP (stretches between Greece, Albania, and Italy). This chain of infrastructure projects of the Southern Gas Corridor directly connects natural gas fields in the Cbaaspian Sea to EU markets. In 2021, Azerbaijani export of natural gas through the Southern Gas Corridor was 19 bcm, 8.5 bcm to Turkey, almost 7 bcm to Italy and the rest to Georgia, Greece, and Bulgaria (Mehdiyev 2022). The project has the potential for substantial expansion and the development of Trans-Caspian energy connectivity between Turkmenistan and Azerbaijan trough the Trans-Caspian Pipeline (TCP), which could tap Turkmenistan’s gas reserves, eventually deliver them via Georgia and Turkey to the EU and would be a major step forward.

The development of the Georgian portion of the Transcaspian International Transport Corridor (TITR), sometimes referred to as the “middle corridor” within China’s Belt and Road initiative, constitutes a central agenda for the Georgian government. The Georgia-China relation has been driven by trade and Georgia’s desire to become “an East-West transport hub” (Brattberg et al. 2021). However, as Brattberg et al. (2021) argue “the Middle Corridor – and, therefore, Georgia’s significance as a transit country – is of little geostrategic importance to China^4^. China’s economic engagement in Georgia is rather ad hoc, namely investments from China in Georgia have been welcomed but were not necessarily transformative (Brattberg et al. 2021)^5^. Besides that, “it is unlikely to do anything there that might offend Russia” (Brattberg et al. 2021).

The new Baku–Tbilisi–Kars is part of the Trans-Caspian International Transport Route (TITR), a much wider network of rail and sea links between western China and Europe. It has the capacity to carry 6.5 million tons of freight per year, a figure which is expected to eventually increase to 17 million tons by 2034 (MFA Turkey 2022). Freight traffic has been increasing steadily on the line since it began operating in late 2017. Georgia offers both land and sea transport routes that facilitate transportation along the corridor. First, it provides an overland route to Turkey, especially via the BTK. Second, Georgia can serve as a maritime outlet to Europe via its port in the Black Sea. In particular, the movement of containers along the middle corridor from China to Georgia increased by 12.9% in 2020 compared to 2019, and this figure increased by 260% in the first half of 2021. As Brattberg et al. (2021) note, “so-called block trains, which operate from origin to destination with all documentation having been arranged in advance, are also now being used on this route.” In 2021, a block train took only 21 days to travel from Xian in China to Tbilisi (Business Media Georgia 2021).

As a part of the post-war settlement over Karabakh, a potentially significant development for Georgia may be the re-opening of the direct railway line between Azerbaijan, Armenia, and Turkey. When a new southern route is fully implemented, this route may attract some volumes from Central Asia, which means they will bypass Georgian Black Sea ports, as well as some volumes could be redirected from the Baku-Tbilisi-Kars (BTK) railway toward the Baku-Nakhchivan-Turkey direction. A fall in traffic along the BTK will mean revenue losses for Georgia.

Georgia’s tourism sector (mountains and sea resorts) has experienced continuous growth and diversification, becoming one of the fastest growing economic sectors in the country before the Covid-19 pandemic. Besides, its development is interconnected with several other sectors of the economy, such as transport and infrastructure. The tourism sector made up 7.5% of GDP growth from 2018–2019 and Georgia received “a record number of 9.3 million international visitors in 2019”, which represents an annual growth rate of 7% (Trade.Gov 2021). Under the top ten source countries were three Black See countries: Russia (20%), Turkey (16%) and Ukraine (2.2%) (Georgian National Tourism Administration 2020).

Georgia is focusing efforts on upgrading the East–West highway, which connects Azerbaijan and the Black Sea coast across Georgian territory, as well as modernizing its freight train network. All these projects are explicitly designed to improve Georgia’s transportation capacity. Georgia’s ‘soft’ infrastructure for trade facilitation and logistics compares well with others in the Central Asia and South Caucasus region, but there are clearly significant gaps that need to be addressed (The World Bank 2020)^6^. Furthermore, still relatively poor transport infrastructure and quality of logistics hamper integration with external markets as well as internal connectivity in Georgia.

## Future of the Black Sea Area and the role of the EU

The Black Sea Area represents a complex geographic space loaded with geopolitical and geo-economic rivalries, which offers both risks and opportunities to its littoral states. As discussed in this chapter, the main security risks for Georgia emanate from Russia’s geopolitical presence in the BSR and Kremlin’s increasing assertiveness against the pro-Western states of the region. Georgia’s security and stability remains highly vulnerable due to the presence of unresolved conflicts, Russia’s continued destabilization measures as well as potential spill overs from an unstable neighborhood.

The Western actors can support Georgia, boost its security and promote peace and stability in a wider Black Sea area. For this, the EU and its member states could develop their own security arrangements, as a way to ensure sustainable peace, stability, and prosperity in the region. In practical terms, this could mean expanding the topics of Strategic Security Dialogue between the EU and Georgia, scaling up support from the European Peace Facility, expanding the EUMM mission, furthering the expansion of Georgia’s already solid participation in various CSDP missions, including Georgia in PESCO projects, supplying defensive military equipment, and including Georgia’s security-related concerns in the dialogue with Russia.

Similarly, NATO-Georgia cooperation needs to intensify even if below membership threshold. In accordance with its new approach, the NATO can strengthen Georgia’s security resilience by boosting its military capacity and civil preparedness. Georgia has to strengthen bilateral strategic partnership with important actors such as Turkey, Ukraine, UK, and US. Georgia should especially aim at further improvement of its highly advanced strategic partnership with the US, with the final long-term objective of becoming its major non-NATO ally in case Georgia’s NATO membership prospects continue to hang in limbo.

Next to security challenges, the BSR can also generate negative externalities for Georgia’s economic development and energy security as well as undermine the country’s transit and connectivity potential. The EU-promoted new regional infrastructure and investment projects have the potential to play a key role in Georgia’s economic development, boosting its connectivity and transit functions and strengthening resilience against domestic and external risks. Against this background, the development of strategic connectivity in the region is in best interest of both the EU and Georgia, and would play an important role in providing alternative routes for energy, transportation, trade, and data connection to Western markets. In 2018, the EU adopted its own strategy to connect Europe and Asia intending to foster a network of rail, road, and maritime routes; and Georgia’s seeks to be part of this endeavor.

With a new connectivity strategy, the EU is starting to play a more active role in shaping the rules around the connectivity in the eastern neighborhood and beyond. During this decade Georgia counts on funds allocated for extension of Trans-European Transport Network to the EaP countries. If successfully implemented, projects will significantly improve Georgia’s physical and digital connections with EU countries on the western shore of the Black Sea (Agenda.ge 2021).

To ensure the stability and cooperative growth in the Black see region, it is in the EU’s interest to boost presence and show stronger leadership. As a mediating power the EU could help coordinate positions and various interests in order to encourage much-needed regional economic integration. In turn, closer economic association will enable all countries involved to withstand Russia’s persistent economic coercion as well as to balance China’s growing economic influence in the region.

## Footnotes

1 Many in Georgia think the EU avoided calling Georgia “European country” not to invoke any association with Article 49 of the EU treaty. See: Chkhikvadze (2019).

2 Overall, in recent years from 10 to 11 million tons of goods transited through Georgia a year by railway, and from 6 to 7 million went by road. See: Lomsadze (2021).

3 Natural gas is the largest energy source in Georgia’s energy consumption, accounting for 36.4% in 2020. According to Energy Balance of Georgia the share of gas from Russia constituted 0.7% in the total supplies in 2018 (99.3% from Azerbaijan), but this figure increased to 17% (83% from Azerbaijan) as compared to 2021. See: Bakradze (2022).

4 In 2020, it was estimated that 81.5% of Chinese cargo (more than 10 million TEU) was shipped by sea, with most of the rest going via the northern route (through Russia and into Central Europe). As little as 1% of cargo travelled via the Middle Corridor. See: Brattberg et al. (2021).

5 According to a public opinion poll commissioned by IRI in February 2021, Georgian citizens think that China is neither a strong friend nor a threat. Moreover, even though China and Georgia have dynamic trade and economic ties, only 11% of Georgian respondents consider China to be Georgia’s “most important economic partner.” See: International Republican Institute (2021).

6 According to the Liner Shipping Connectivity Index 2020, the country is especially lagging behind in the efficiency of seaport and transport services, the quality of roads, airport connectivity, logistics quality and competence, and tracking and tracing. See: European Investment Bank (2021).
